# Intrastromal misplacement of a foldable IOL during phacoemulsification: Intraoperative OCT identification of type-1 dua's layer separation

**DOI:** 10.1016/j.ajoc.2026.102578

**Published:** 2026-04-07

**Authors:** Haruka Ueda, Akira Kobayashi, Natsuko Mori, Hideaki Yokogawa, Tomomi Higashide

**Affiliations:** Department of Ophthalmology, Kanazawa University Graduate School of Medical Science, Kanazawa, Japan

**Keywords:** Intrastromal intraocular lens, Dua's layer, Type-1 separation, Anterior segment OCT, Cataract surgery complication

## Abstract

**Background:**

Intrastromal misplacement of a foldable intraocular lens (IOL) during cataract surgery is exceedingly rare. Immediate identification of the separation plane is critical to prevent additional corneal injury.

**Case report:**

A 76-year-old woman with bilateral glaucoma underwent routine phacoemulsification. During insertion of a one-piece acrylic IOL, the lens was inadvertently delivered into the corneal stroma. The IOL remained partially unfolded within a stromal pocket. Intraoperative anterior segment optical coherence tomography (AS-OCT; RESCAN 700) clearly demonstrated deep stromal migration of the IOL and a thick, taut, hyperreflective line consistent with a Type-1 separation of Dua's layer adherent to Descemet's membrane. This real-time confirmation enabled controlled removal of the misdirected IOL without Descemet's membrane rupture, followed by successful in-the-bag implantation of a new IOL and intracameral air tamponade. Postoperative AS-OCT showed gradual resolution of the separation without additional intervention. At 6 months, the cornea remained clear with improved visual acuity (0.6 to 0.7; logMAR 0.22 to 0.15) and an endothelial cell density of 1253 cells/mm^2^ (52.8% loss). At 17 months, visual acuity remained 0.7, and endothelial cell density was 1158 cells/mm^2^ (56.4% loss), and corneal clarity was maintained.

**Conclusions:**

Intraoperative AS-OCT provided decisive, real-time visualization of the stromal entry plane and Type-1 Dua's layer separation, allowing safe retrieval of the misdirected IOL and preservation of Descemet's membrane integrity. Recognition of this separation pattern is essential for guiding atraumatic management of this extremely rare complication.

## 1 Background

Iatrogenic corneal complications during cataract surgery are uncommon but may significantly affect postoperative outcomes. Among these, intraoperative misplacement of an intraocular lens (IOL) into the corneal stroma is particularly rare. Since Dua et al. first described Dua's layer in 2013^1^—a thin, compact, acellular layer situated between the posterior stroma and Descemet's membrane—attention has increased toward posterior corneal pathologies potentially involving this plane.

Although the existence and clinical relevance of Dua's layer remain debated,^2^ differentiation of posterior corneal separation patterns may have practical implications in surgical settings. Posterior corneal separation can be broadly categorized into Type-1, in which Dua's layer remains adherent to Descemet's membrane, and Type-2, in which Descemet's membrane alone is detached. Several reports have described inadvertent IOL entry into the pre-Descemet or posterior stromal space 3, 4, 5, 6, 7. In these cases, postoperative AS-OCT findings such as stromal separation^4^ and corneal thickness changes^5^ have been documented; however, real-time intraoperative AS-OCT visualization of the separation plane at the moment of IOL misplacement has not previously been reported.

Herein, we present a case in which intraoperative AS-OCT revealed morphological features consistent with a Type-1 separation involving Dua's layer, as defined by Dua et al.,^2^ during accidental intrastromal IOL misplacement. A schematic illustration of Type-1 and Type-2 posterior corneal separation patterns is shown in Fig. 3.

## 2 Case report

This study adhered to the tenets of the Declaration of Helsinki, and written informed consent was obtained for publication of clinical information and images.

**Fig. 1:**
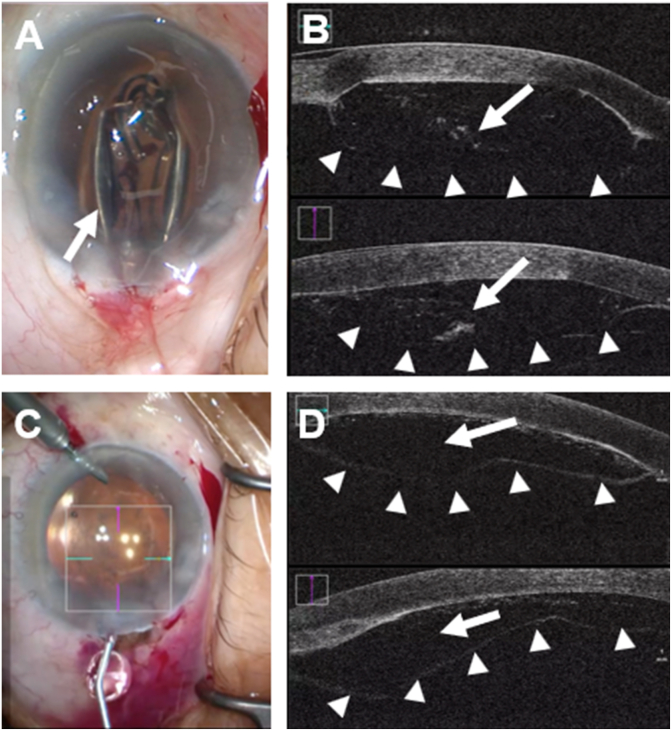
Intraoperative findings of intrastromal misplacement of an intraocular lens (IOL). (A) Intraoperative microscope view showing the misdirected, partially unfolded IOL (arrow). (B) Intraoperative AS-OCT demonstrating the IOL located within the posterior corneal stroma (arrow). The separated layer appears as a thick, taut hyperreflective line (arrowheads), morphologically consistent with a Type-1 separation, with Dua's layer adherent to Descemet's membrane. In contrast, Type-2 separation appears as a thin, undulating contour, representing detachment of Descemet's membrane alone. (C) Removal of the misplaced IOL using Shephard lens forceps. (D) Intraoperative AS-OCT after IOL removal showing a residual posterior stromal separation pocket (arrow). The arrowheads indicate the separated posterior corneal layer (Type-1 separation).

A 76-year-old woman with bilateral glaucoma controlled medically presented with progressive visual decline from cataracts. Preoperative best-corrected visual acuity (BCVA) was 0.6 (logMAR 0.22) in the right eye, with an endothelial cell density (ECD) of 2657 cells/mm^2^. Standard phacoemulsification was performed through a 2.4-mm temporal clear corneal incision. During implantation of a preloaded one-piece acrylic IOL (DCB00V; Johnson & Johnson Vision), the injector tip did not fully enter the anterior chamber due to a tight incision. As a result, the IOL was inadvertently delivered into the corneal stroma and remained partially unfolded within a stromal pocket. Intraoperative anterior segment optical coherence tomography (AS-OCT; RESCAN 700) demonstrated deep stromal migration of the IOL and a distinct, taut, hyperreflective line consistent with a Type-1 separation at the level of Dua's layer (Fig. 1A and B). Based on this real-time morphological information, the main incision was enlarged, and the misdirected IOL was carefully retrieved with lens forceps (Fig. 1C and D). After retrieval of the misdirected IOL, the intrastromal interface was gently irrigated with balanced salt solution (BSS) to remove residual material. A new one-piece acrylic IOL was subsequently implanted in the capsular bag using the standard injector system. An intracameral air bubble was placed to tamponade the separation plane, and the wound was secured with nylon sutures.

**Fig. 2:**
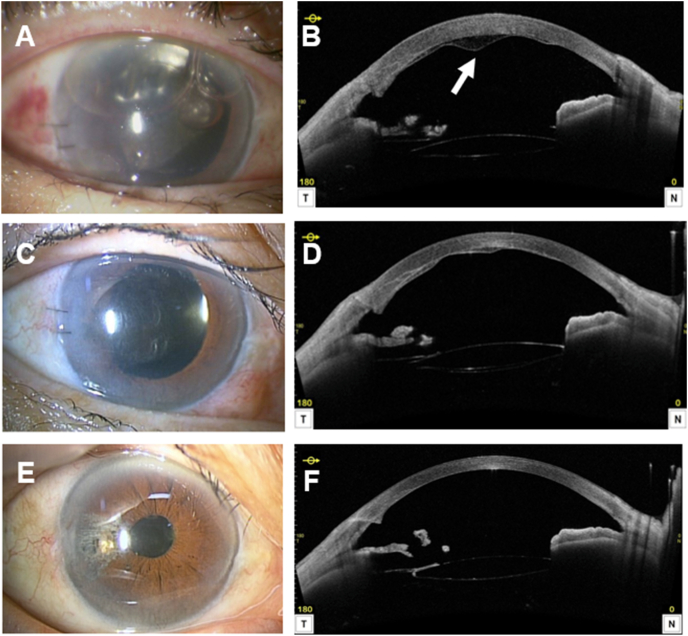
Postoperative course of corneal recovery on slit-lamp biomicroscopy and AS-OCT (CASIA2). (A, B) Postoperative day 1 showing corneal edema and mild stromal separation (arrow). (C, D) Day 8 showing reduction of corneal edema and partial reattachment (arrow). (E, F) Month 17 showing clear cornea and complete reattachment. Endothelial cell density decreased from 2657 cells/mm^2^ preoperatively to 1158 cells/mm^2^ (56.4% loss) at 17 months, but the cornea remained clear with visual acuity of 0.7 (logMAR 0.15).

Postoperatively, mild corneal edema resolved within one week. Serial AS-OCT (CASIA2) demonstrated gradual re-approximation of the separated layer and restoration of posterior stromal architecture (Fig. 2). At 6 months, the cornea remained clear with improved BCVA of 0.7 (logMAR 0.15) and an ECD of 1253 cells/mm^2^ (52.8% loss). At 17 months, visual acuity remained stable at 0.7, and the ECD was 1158 cells/mm^2^ (56.4% loss), with persistent corneal clarity.

**Fig. 3:**
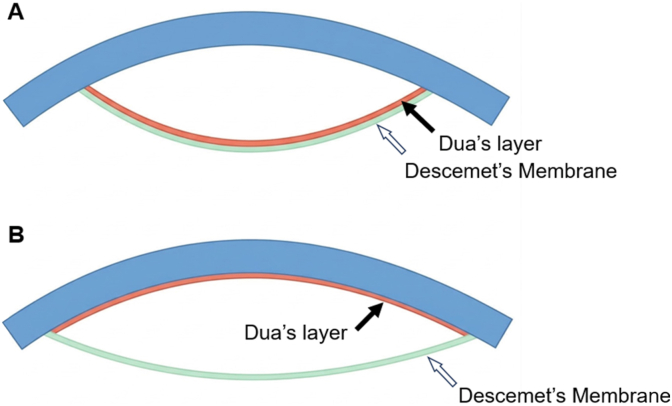
Schematic illustration of posterior corneal separation patterns. (A) In Type-1 separation, Dua's layer (black arrow) remains adherent to Descemet's membrane (white arrow), and both layers detach together from the posterior stroma as a thick, compact, taut unit. (B) In Type-2 separation, only Descemet's membrane (white arrow) detaches from the posterior stroma, while Dua's layer (black arrow) remains adherent to the stroma, resulting in a thinner and more fragile configuration.

## 3 Discussion

Intrastromal misdirection of an intraocular lens (IOL) during cataract surgery is exceptionally rare. Previous reports have generally described lens entry into the pre-Descemet or posterior stromal space 3, 4, 5, 6, 7, with postoperative anterior segment optical coherence tomography (AS-OCT) used to document stromal separation or corneal thickness changes 3, 4, 5, 6. However, none have provided real-time intraoperative AS-OCT characterization of the actual separation plane at the moment of misplacement, underscoring the novelty of the present case (Table 1).

**Table 1:** Comparison of reported cases of intraocular lens misplacement into the corneal stroma.

Study	Year	Age/Sex	IOL Type	Technique	AS-OCT Timing	Dua's Layer Classification	Endothelial Loss	Final BCVA	Reference
Present case	2026	76/F	One-piece acrylic (DCB00V)	Injector	Intraoperative	Type-1 separation	56.4% (2657 → 1158 cells/mm^2^, at 17 months postoperatively)	0.7 (logMAR 0.15)	-
Haddad et al.	2025	75/M	Preloaded acrylic	Wound-assisted	Postoperative	Not specified	NR	0.66 (logMAR 0.18)	3
Çukurova et al.	2024	72/M	One-piece acrylic (SA60AT)	Wound-assisted	Postoperative	Not specified	21.4% (2257 → 1773 cells/mm^2^, at 2 months postoperatively)	1.0 (logMAR 0)	4
Toker et al.	2024	67/F	One-piece acrylic (Zaraccom F260)	Wound-assisted	Not performed	Not specified	NR	0.5 (20/40)	5
Moriya et al.	2021	84/F	One-piece acrylic (SN6AT3)	Wound-assisted	30 minutes postoperative	Not specified (Thin line)	No significant loss (2817 → 2941 cells/mm^2^ at 4 days postoperatively)	1.0 (logMAR 0)	6
Shah et al.	2014	60/M	Foldable acrylic	Injector	Postoperative	Not specified	NR	0.5 (20/40)	7

AS-OCT = anterior segment optical coherence tomography; BCVA = best-corrected visual acuity; F = female; IOL = intraocular lens; M = male; NR = not reported.

In this patient, the misdirection occurred not during a wound-assisted technique but during standard preloaded injector insertion, in which the injector tip failed to fully enter the anterior chamber. As a result, the IOL was delivered into the posterior stroma rather than the anterior chamber.

When a tight corneal incision prevents full insertion of the injector tip into the anterior chamber, the tip may become engaged at the level of the posterior limiting lamina (Dua's layer). If the IOL is advanced while the tip remains partially embedded at this level, shear forces can be generated along this plane, leading to intrastromal misdirection of the IOL and resulting in a Type-1 separation, as observed in the present case. To prevent this complication, surgeons should ensure that the injector tip is fully inserted into the anterior chamber before advancing the IOL. If resistance is encountered at the incision, the wound should be appropriately enlarged rather than forcing the injector forward.

Intraoperative AS-OCT captured a thick, taut, hyperreflective line, consistent with a Type-1 separation as described by Dua et al.^2^ In Type-1 separation, Dua's layer remains attached to Descemet's membrane and separates together as a compact unit from the posterior stroma, whereas Type-2 separation involves Descemet's membrane alone and is much thinner and more fragile. Although histologic confirmation was not possible, the intraoperative imaging provided strong evidence that the IOL entered a deep posterior stromal cleavage plane compatible with a Type-1 configuration. This information was clinically meaningful, as it reassured the surgeon that controlled removal was feasible without risking immediate membrane rupture.

In contrast, a true Type-2 separation—where only Descemet's membrane detaches—is unlikely to permit temporary placement of an IOL due to its low tensile strength. To date, no cases of intrastromal IOL misplacement associated with a Type-2 pattern have been reported.

The present case also demonstrates the practical value of intraoperative AS-OCT for guiding safe management. By confirming the depth and morphology of the separation, the surgeon was able to retrieve the misdirected IOL with minimal additional trauma and to apply an intracameral air bubble to reapproximate the separated plane, consistent with previous reports 3, 4, 5, 6, 7.

Air tamponade was selected to promote reapposition of the separated posterior stromal layers, consistent with its established role in Descemet stripping automated endothelial keratoplasty (DSAEK) and related procedures. Air provides uniform posterior pressure, facilitating adhesion of the posterior limiting lamina (Dua's layer) and Descemet's membrane to the underlying stroma. In contrast, the use of viscoelastic materials (OVDs) was not favored, as their tamponade effect is generally weaker and cohesive OVDs in particular may increase the risk of postoperative intraocular pressure elevation. In addition, retained OVD within the interface could potentially delay reattachment. Although additional measures such as venting incisions for active interface drainage may be considered, they were not performed in this case, as spontaneous resolution of residual interface fluid was anticipated under air tamponade.

Despite the favorable anatomical recovery, the patient experienced significant endothelial cell loss (a reduction of 52.8% at 6 months and 56.4% at 17 months). The acute endothelial injury—primarily attributable to mechanical trauma, intraoperative endothelial contact during IOL manipulation, and air bubble–endothelium interaction—appears to have been the main driver of this marked ECD reduction. Notably, the additional decrease in ECD between 6 months (1253 cells/mm^2^) and 17 months (1158 cells/mm^2^) was relatively small (95 cells/mm^2^; approximately 7.6% over 11 months), suggesting that the acute injury phase had largely subsided by 6 months and that endothelial redistribution had reached a plateau or a slower decline phase thereafter. Although chronic topical glaucoma medications may have contributed to baseline endothelial vulnerability, they are unlikely to explain the acute magnitude of cell loss observed. Although the remaining cell density (1158 cells/mm^2^) is above the critical threshold for decompensation, careful long-term monitoring is warranted given the substantial overall reduction and the patient's glaucoma with chronic topical medication use, due to the potential risk of late-onset corneal decompensation.

This case highlights the broader surgical relevance of posterior stromal anatomy and suggests that intraoperative AS-OCT may help distinguish different posterior corneal planes in real time. Such information may be valuable not only in rare complications like this one but also in procedures involving Descemet's membrane manipulation, including endothelial keratoplasty and deep anterior lamellar keratoplasty. To prevent intrastromal IOL misplacement, surgeons should ensure full insertion of the injector nozzle into the anterior chamber and maintain stable chamber depth during lens delivery.

Several limitations should be acknowledged. First, the suspected involvement of Dua's layer is based on imaging morphology rather than histopathologic confirmation. Second, the mechanism of ongoing endothelial loss remains uncertain and may involve multiple contributing factors. Third, as a single case, the reproducibility of the management strategy and the generalizability of the observed endothelial decline cannot be determined. Lastly, confocal microscopy was not performed, which may have provided further insights into endothelial health.

## 4 Conclusions

This case demonstrates that intraoperative OCT can provide real-time visualization of a separation plane consistent with Type-1 Dua's layer involvement during intrastromal IOL misplacement. Immediate imaging enabled precise localization, safe removal of the misdirected IOL, and effective air tamponade, resulting in favorable anatomical and visual outcomes. Surgeons should consider immediate OCT assessment when abnormal lens unfolding is encountered during cataract surgery.

## CRediT authorship contribution statement

**Haruka Ueda:** Investigation, Data curation. **Akira Kobayashi:** Writing – review & editing, Supervision. **Natsuko Mori:** Investigation, Formal analysis. **Hideaki Yokogawa:** Writing – original draft, Data curation, Conceptualization. **Tomomi Higashide:** Supervision, Funding acquisition, Data curation.

## Patient consent

Written informed consent to publish this case report, including clinical data and images, was obtained from the patient.

## Authorship

All authors attest that they meet the current ICMJE criteria for Authorship.

## Use of artificial intelligence

The authors used AI-assisted language editing tools (ChatGPT, developed by OpenAI; https://openai.com) solely for grammar and spelling correction of the English text. AI tools were not used for data analysis, scientific interpretation, clinical decision-making, or generation of any scientific content. All authors have reviewed and approved the final manuscript and take full responsibility for its accuracy and integrity.

## Funding

No funding or grant support.

## Declaration of competing interest

The authors declare that they have no known competing financial interests or personal relationships that could have appeared to influence the work reported in this paper.
